# CRISPR/Cas9-mediated SNAC9 mutants reveal the positive regulation of tomato ripening by SNAC9 and the mechanism of carotenoid metabolism regulation

**DOI:** 10.1093/hr/uhad019

**Published:** 2023-02-10

**Authors:** Yuan Feng, Xiaohong Kou, Shuai Yuan, Caie Wu, Xiaoyang Zhao, Zhaohui Xue, Qingxiu Li, Zhengyu Huang, Yijie Sun

**Affiliations:** School of Chemical Engineering and Technology, Tianjin University, Tianjin 300072, China; School of Chemical Engineering and Technology, Tianjin University, Tianjin 300072, China; School of Chemical Engineering and Technology, Tianjin University, Tianjin 300072, China; College of Light Industry and Food Engineering, Nanjing Forestry University, Nanjing 210037, China; School of Chemical Engineering and Technology, Tianjin University, Tianjin 300072, China; School of Chemical Engineering and Technology, Tianjin University, Tianjin 300072, China; College of Food Science and Biological Engineering, Tianjin Agricultural University, Tianjin 300384, China; School of Chemical Engineering and Technology, Tianjin University, Tianjin 300072, China; School of Chemical Engineering and Technology, Tianjin University, Tianjin 300072, China

## Abstract

NAC transcriptional regulators are crucial for tomato ripening. Virus-induced gene silencing (VIGS) of *SNAC9* (*SlNAC19*, Gene ID: 101248665) affects tomato ripening, and *SNAC9* is involved in ethylene and abscisic acid (ABA) metabolic pathways. However, the function of *SNAC9* in pigment metabolism in tomatoes remains unclear. This work seeks to discover the mechanism of *SNAC9* involvement in pigment metabolism during tomato ripening by establishing a *SNAC9* knockout model using CRISPR/Cas9 technology. The results indicated that fruit ripening was delayed in knockout (KO) mutants, and *SNAC9* mutation significantly affected carotenoid metabolism. The chlorophyll (Chl) degradation rate, total carotenoid content, and lycopene content decreased significantly in the mutants. The transformation rate of chloroplasts to chromoplasts in mutants was slower, which was related to the carotenoid content. Furthermore, *SNAC9* changed the expression of critical genes (*PSY1*, *PDS*, *CRTISO*, *Z-ISO*, *SGR1*, *DXS2*, *LCYE*, *LCYB*, and *CrtR-b2*) involved in pigment metabolism in tomato ripening. *SNAC9* knockout also altered the expression levels of critical genes involved in the biosynthesis of ethylene and ABA. Accordingly, *SNAC9* regulated carotenoid metabolism by directly regulating *PSY1*, *DXS2*, *SGR1*, and *CrtR-b2*. This research provides a foundation for developing the tomato ripening network and precise tomato ripening regulation.

## Introduction

In recent years, the loss and waste of horticultural crops after harvest have exacerbated agricultural problems [1]. One significant and nutrient-rich horticultural crop is tomato (*Solanum lycopersicum*). The complex physiological and biochemical process of tomato ripening includes pigment accumulation, fruit softening, aroma, and flavor development [2]. In addition, the peak respiration rate and ethylene release values co-occurred within the climacteric fruit [3]. Tomato is a typical model for climacteric fruit research [4].

The degradation of Chl and the accumulation of pigments such as carotenoids change the color of tomatoes [5]. Carotenoids are closely related to human health, providing precursors for synthesizing vitamin A, effectively preventing night blindness, improving immunity, and delaying cardiovascular disease [6]. There are at least 12 types of carotenoids in tomato fruits, including lycopene, β-carotene, lutein, ζ-carotene, α-carotene, γ-carotene, and δ-carotene. Geranylgeranyl pyrophosphate (GGPP) is first produced before carotenoids are biosynthesized via the methylerythritol 4-phosphate (MEP) pathway. GGPPs are then compressed by phytoene synthase (PSY) to produce phytoene. Phytoene desaturase (PDS), ζ-carotene desaturase (ZDS), carotenoid isomerase (CRTISO), and ζ-carotene isomerase (Z-ISO) all work together to create lycopene. α-carotenoids, lutein, β-carotenoids, zeaxanthin, violaxanthin, and neoxanthin are produced by the enzymes lycopene ε-cyclase (LCYE) and lycopene β-cyclase (LCYB) [7]. In fruit tissue, carotenoids are isolated in the chromoplast, and ultrastructural changes in the chromoplast are related to carotenoids [8, 9].

Carotenoid production is closely controlled at both the transcriptional and post-transcriptional levels [10, 11]. A study found that *SlZHD17* directly regulated the carotenoid biosynthesis genes *SlPSY1* and *SlZISO* [12]. *SlHY5* is involved in tomato ripening through the transcriptional regulation of carotenoid biosynthesis [13]. *SlIDI1* deficiency blocked carotenoid synthesis [11]. The NAC family, named after NAM, ATAF1/ATAF2, and CUC2, is one of the largest families of TFs that plays an essential role in plant development and fruit ripening at different stages. Several studies have shown that the NAC transcription factors regulate carotenoid metabolism [14, 15]. *SlNAC4* and *SlNOR-like1* are positive regulators of tomato pigment formation [16, 17]. The promoters of several genes involved in color change are directly bound by NOR-like1 [18]. During tomato ripening, ethylene regulates color changes, including Chl reduction and biosynthesis of carotenoids or anthocyanins [19, 20]. By reducing the expression of *SlPSY1*, *SlACO1*, *SlACS2*, and *SlACS4*, SlNAC1 regulates fruit ripening through ethylene- and ABA-dependent mechanisms [21]. *SlNAM1* is also involved in fruit ripening and ethylene production [22]. Our earlier research demonstrated that NAC transcription factors are crucial for ethylene production, reception, and signaling [23]. Tomato fruit ripening is influenced by *SNAC4/9* synergistically by influencing ethylene and ABA metabolisms [24].

CRISPR/Cas9 technology is crucial to understanding how NAC regulates fruit ripening theoretically and practically. Defects such as sterility, self-incompatibility, excessive heterozygosity, a lack of alleles and features necessary for recovery, and a long life cycle can all be avoided by CRISPR [1, 25]. Several crops, including tomatoes, *Arabidopsis*, rice, and soybeans, have benefited from the induction of beneficial characteristics using CRISPR/Cas9 mutations [26, 27]. In tomatoes, several ripening-related CRISPR/Cas9-mediated regulatory mutants have been identified [28, 29]. Gao et al. [16, 22] studied *SlNAM1*-deficient and *SlNOR-like1* mutants with CRISPR/Cas9 technology.

**Figure 1:**
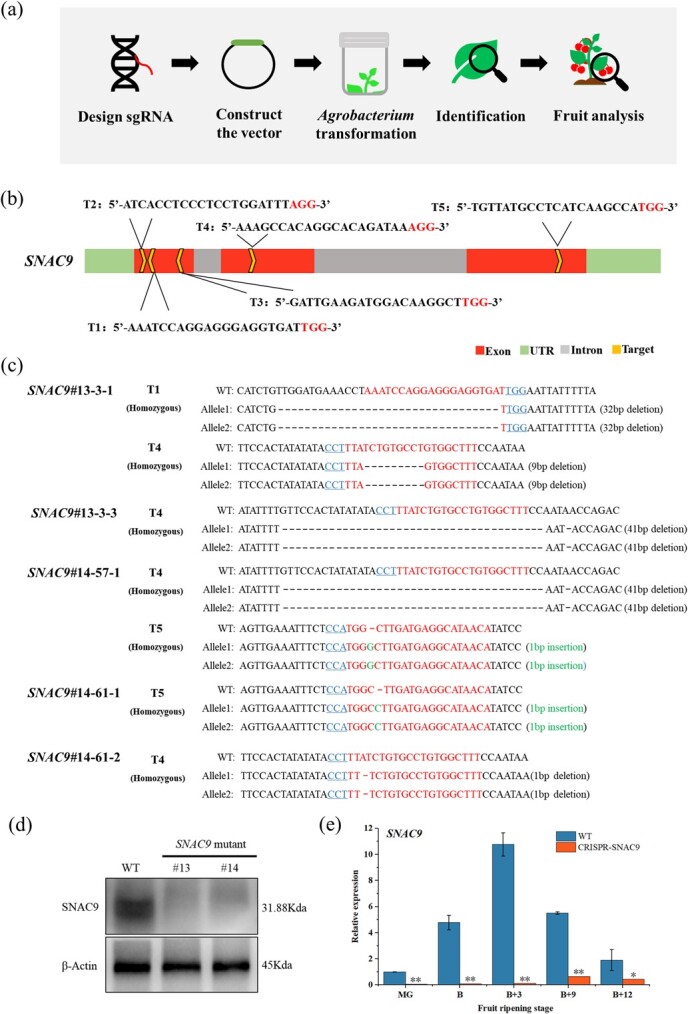
Identification of CRISPR/Cas9-*SNAC9* knockout mutants. (a) Gene editing process. (b) Five target locations in the *SNAC9* gene sequence are shown schematically. (c) Sequencing results of the T_1_ generation mutant. The target sites are indicated by red lettering. The protospacer adjacent motif (PAM) is represented by blue letters. Black “-” represents deletion. Green bases represent insertion. (d) The expression of SNAC9 protein in SNAC9#13 and SNAC9#14 mutants was detected by Western Blot. (e) The relative expression level of the *SNAC9* gene at different ripening stages. Tomato *Actin* gene was used for the internal reference gene. MG, mature green. B, breaker. B + 3, breaker+3 days. For each analysis, three biological replicates (n = 3) were used. The bars represent the averages and standard deviations of 3 biological replicates. The standard deviations are shown by the error bars. Asterisks show significant differences between WT and *SNAC9*-KO fruit (^*^P ≤ 0.05, ^**^P ≤ 0.01).

In previous studies, VIGS of *SNAC9* reduced lycopene accumulation and delayed the ripening of tomato fruits. Silencing the *SNAC4* and *SNAC9* genes resulted in opposite changes in ABA content and fruit softening rate in tomato fruits [24], and the regulation of pigment changes was consistent. However, uncertainty still exists regarding how pigment metabolism is regulated throughout the ripening process of tomato fruits. In this study, we eliminated the *SNAC9* gene at both single and multiple sites using CRISPR/Cas9 technology (Fig. 1a), mainly to investigate how *SNAC9* affects the metabolism of tomato carotenoids and the target gene of *SNAC9* that regulates tomato pigment metabolism. We verified that *SNAC9* positively regulated carotenoid production in tomatoes. This study lays a foundation for establishing a tomato ripening regulatory network.

## Results

### Construction and identification of CRISPR/Cas9-*SNAC9* knockout mutants

Studies have demonstrated that the CRISPR/Cas9 system may generate homozygous mutants in diploid plants such as rice, tomato, and *Arabidopsis* [28, 30]. Mutations at different gene sites have different effects on protein function. We designed five *SNAC9* knockout targets in three exons of *SNAC9* (Fig. 1b) and constructed a single guide RNA (sgRNA) expression cassette through overlapping PCR. Using Golden Gate cloning, single or multiple sgRNA expression cassettes were combined into a pYLCRISPR/Cas9 vector. PCR and sequencing results showed that the *SNAC9* knockout vector was successfully constructed. The genes encoding Cas9 protein and sgRNA were introduced into Micro-Tom tomato cells by the *Agrobacterium* transformation method, and target- and off-target-positive seedlings were detected. The results showed that tomatoes were mutated successfully without any off-target effects. The CRISPR/Cas9 vector system in this study demonstrated excellent specificity in tomatoes.

The sequencing results at the target and the wild-type (WT) sequences showed that 26 mutants were successfully identified. Typical sequencing results of the T_1_ generation are shown in Fig. 1c. Ten strains were double-target mutations, and it is worth noting that large fragment sequences were lost in the T1 and T4 targets of 13–3. These results indicated that the target modification sites of the CRISPR/Cas9 gene editing tomato were inherited stably between generations. *SNAC9* has transcriptional regulatory functions [31]. *SNAC9* knockout reduced the average expression level of *SNAC9* by 96.7% in five stages (Fig. 1e), indicating that the *SNAC9* gene was knocked out successfully with high knockout efficiency. Western blot analysis revealed that the WT but not the #13 and #14 mutants expressed the SNAC9 protein (Fig. 1d). We verified the successful construction of SNAC9 knockout mutants at the gene and protein levels.

### Tomato fruit ripening is delayed in *SNAC9* CRISPR/Cas9-edited lines

**Figure 2:**
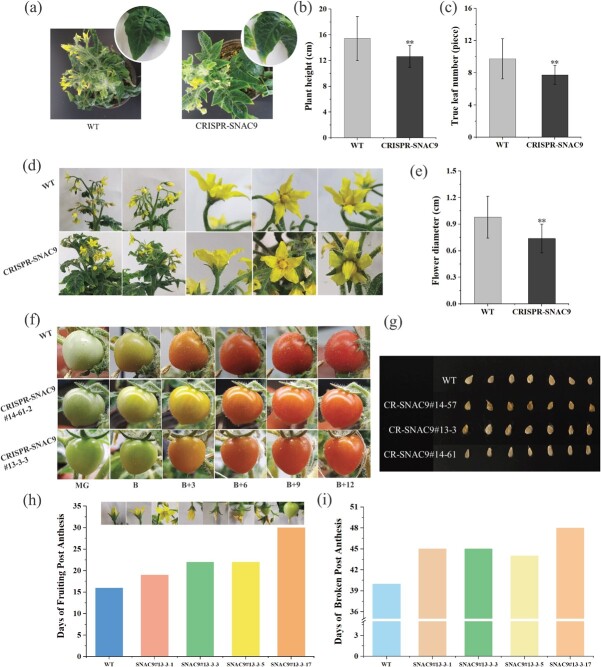
The ripening of tomato fruit was significantly delayed in *SNAC9* mutants. (a) Plant phenotypic. (b) Plant height. (c) True leaf number. (d) Flower phenotype. (e) Flower diameter. Error bars indicate the mean ± SD of at least 15 biological replicates. Asterisks show significant differences between WT and *SNAC9*-KO fruit (^**^P ≤ 0.01). (f) The ripening of tomato fruit was noticeably delayed in *SNAC9*#14 and *SNAC9*#13 mutants. (g) The seed was morphological. (h) Time of fruiting after flowering. (i) Color breaking time after flowering was delayed in *SNAC9* mutants.

To ascertain whether gene knockout of *SNAC9* affected tomato fruit ripening, fruit setting time, breaker time, fruit development period, and ripening phenotype were documented (Fig. 2). The mutant had much lower plant height, true leaf number, and blossom diameter than the WT (Fig. 2a-e). The post-flowering fruition and color-breaking time of the KO line were delayed (Fig. 2h-i). Fruit phenotypic changes were recorded in six periods according to the days after the breaker stage (Fig. 2f). Fruit color development was delayed in the *SNAC9* KO line at the B + 3 stage. WT fruit is already orange, but *SNAC9* knockout fruit is yellow. In the B + 6 stage, WT fruit began to change from orange to red, but the KO line was dark orange. In the B + 12 stage, the wild-type pericarp was entirely red, but the KO line color did not get deeper. In addition, the CRISPR-*SNAC9* #13–3 line had a uniform seed size, similar to that of the WT, and higher plumpness. The CRISPR-*SNAC9* #14–57 line had smaller seeds than the wild type and lower plumpness (Fig. 2g). *SNAC9* gene knockout affects tomato seed development to varying degrees, and different vectors have different effects. These findings suggested that *SNAC9* controls the ripening of tomatoes.

### *SNAC9* affects color and endogenous carotenoid accumulation in tomato fruit

**Figure 3:**
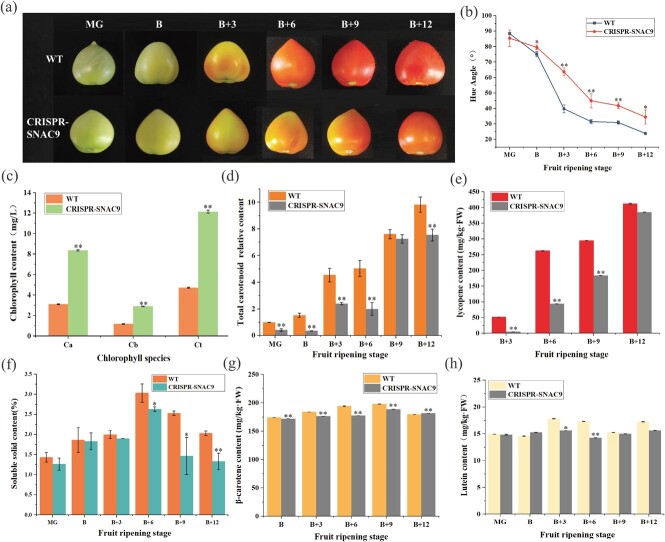
Effect of *SNAC9* on carotenoid accumulation. (a) Fruit color change was delayed in the KO line. (b) Hue angle change. (c) Chlorophyll content. Ca, chlorophyll a. Cb, chlorophyll b. Ct, total chlorophyll. (d) Changes in the relative content of total carotenoids. (e) Lycopene content. (f) Change in soluble solid content. (g) β-carotene content. (h) Lutein content. For each analysis, three biological replicates (n = 3) were used. The bars represent the averages and standard deviations of 3 biological replicates. The standard deviations are shown by the error bars. Asterisks show significant differences between WT and *SNAC9*-KO fruit (^*^P ≤ 0.05, ^**^P ≤ 0.01).

As the fruit ripened, its color substantially altered, signaling the move from the green to the red ripening stage [6], so we measured the changes in fruit hue angle, chlorophyll, carotenoid, and soluble solids. The color angle of the CRISPR-*SNAC9* tomato was substantially higher than that of the WT in the corresponding period from Day 3 to Day 12 of the color breaker stage (Fig. 3a-b). The results indicated that the total amount of chlorophyll a, b, and chlorophyll in the KO line was higher than that in the WT, indicating that the degradation rate of Chl was lower than that in the WT (Fig. 3c). The total carotenoid content of WT fruits was greater than that of CRISPR-*SNAC9* fruits in all six periods (Fig. 3d). At the B + 3 stage, the total carotenoid content of CRISPR-*SNAC9* fruit was 52.79% that of the WT. The total carotenoid content was only 39.58% that of the WT at the B + 6 stage. These results indicate that *SNAC9* significantly affects the regulation of carotenoids. The lycopene content in the *SNAC9*-KO line was lower than that in the WT (Fig. 3e). Lycopene levels were 262.61 mg/kg· FW in WT fruits at the B + 6 stage, but only 93.94 mg/kg· FW in the KO line, which was consistent with the phenotypic observations. The β-carotene content in the KO fruit was lower than that in the WT fruit at the B + 3 stage and then increased to a similar level as that in the WT fruit at the B+ 12 stage (Fig. 3g). The lutein content in the KO fruit was lower than that in the WT fruit at the B + 3 and B + 6 stages (Fig. 3h). According to these findings, *SNAC9* knockout interfered with carotenoid accumulation in tomato fruit.

With the increase in fruit maturity, carbohydrates, cellulose, pectin, and other substances in the fruit were hydrolyzed continuously, and the total amount of respiration metabolism was less than the total amount of degradation, increasing the solid soluble content [24, 32]. WT reached its maximum value in the B + 6 period, reaching 3.03 Brix% (Fig. 3f). CRISPR-*SNAC9* reached its peak in the B + 6 phase, or 2.63 Brix%. After the degradation of macromolecules, the total amount of respiratory metabolism was higher than the total amount of degradation, and the solid soluble content decreased. At the B + 6/9/12 stage, the solid soluble content in the KO line was considerably lower. It was speculated that *SNAC9* knockout restrained the accumulation of lycopene, vitamin C, and organic acids in fruit at the late ripening stage. These substances were positively linearly correlated with the soluble solids content [33].

### *SNAC9* knockout affects ethylene release by regulating ethylene biosynthetic gene expression

The ripening of tomatoes, a climacteric fruit, is intimately correlated with the production of ethylene [20]. The ethylene peak of WT fruits appeared at the B + 3 stage. *SNAC9*-knockout fruits did not exhibit significant heights, and ethylene remained low during ripening (Supplementary Fig. S1). *ACO1*, *ACS2*, and *ACS4* are the most critical ethylene biosynthesis genes [34]. The ethylene synthesis gene expression peak appeared in the control group B + 3 stage (Supplementary Fig. S1), which may be related to the peak of ethylene synthesis. The knockout and wild-type fruits have significantly different *ACO1*, *ACS2*, and *ACS4* expression levels. These results indicated that *SNAC9* gene knockout affected ethylene synthesis by downregulating *ACS2*, *ACS4*, and *ACO1*.

### *SNAC9* regulates ABA biosynthesis and affects pericarp morphology

The ABA content in *SNAC9* knockout and WT tomato fruits is shown in Fig. S2. The ABA content of *SNAC9* mutant fruits was at a high level during ripening and peaked at the B + 3 stage, which was 2.07 times that of WT, and the difference was significant. This was consistent with the regulation of fruit hardness by SNAC9 (Supplementary Fig. S2). During fruit ripening, *SlNCED1/2* are involved in the ABA signaling pathway [35, 36]. Studies have shown that the knockout of the *SNAC9* gene also significantly affects the *NCED* gene (Supplementary Fig. S2). *NCED1* was upregulated in the KO line at the green ripening stage, breaker color stage, and B + 12 stage. In the green ripening stage, B + 3 stage, and B + 9 stage, *NCED2* was upregulated in the KO line. This was consistent with the increased ABA content in the *SNAC9-*KO line.

**Figure 4:**
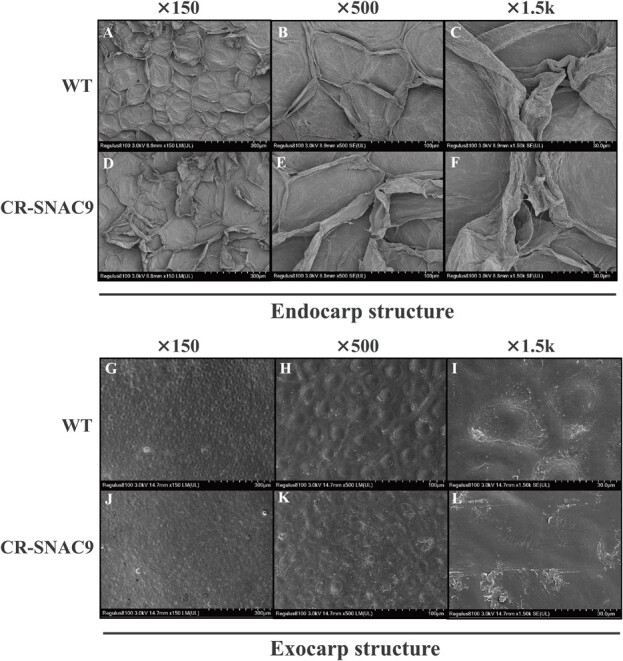
Effects on pericarp structure. Microstructure of the inner and outer pericarp of tomato fruit. A-C, endocarp structure of WT fruit. D-F, fruit endocarp structure in the *SNAC9* KO line. G-I, exocarp structure of WT fruit. J-L, exocarp structure in the *SNAC9* KO line.

The primary cause of fruit softening is cell wall deterioration [2]. WT and CRISPR-*SNAC9* fruit hardness decreased during ripening. Compared to WT fruit within the same period, CRISPR-*SNAC9* fruit had a significantly lower fruit hardness (Fig. 4), and the fruit softening rate was accelerated. It was concluded that *SNAC9* inhibited fruit softening. At the B + 12 stage, tomato endocarp cells exhibited dehydration, cell shrinkage, and cell wall attachment with a filamentous structure. As shown in Fig. 4, WT (A-C) pericarp cells were uniform in size and closely arranged among cells. After observation of the enlarged structure and morphology, WT pericarp cells have a strong three-dimensional shape, the epidermis shape is relatively complete, and the lines are neat. Compared with WT, CRISPR-*SNAC9* (D-F) inner epidermal cells exhibited more cracks, apparent filamentous structure attached to the cell wall, severe water loss, and cell shrinkage. This was associated with reduced fruit hardness in the *SNAC9* KO line. The cell morphology showed apparent differences. The outer epidermis of the WT has a regular structure, with a uniform round protrusion structure, and the cell structure is more closely arranged. At the 1.5 K multiple, there is uniform depression around the protrusion. It was speculated that the accumulation of pectin decomposition enzymes in the fruit ripening process degraded the cell wall, reduced cell plasticity, and resulted in cell relaxation, leading to the collapse of the epidermal structure [37]. However, CRISPR-*SNAC9* cell structure uniformity decreased, the cell center only slightly protruded, cell space significantly increased, and adjacent cell connection tightness decreased. Therefore, it is speculated that *SNAC9* knockout may change the cell morphology during fruit softening.

### *SNAC9* changes the expression of critical genes and chromoplast transformation in tomato pigment metabolism

**Figure 5:**
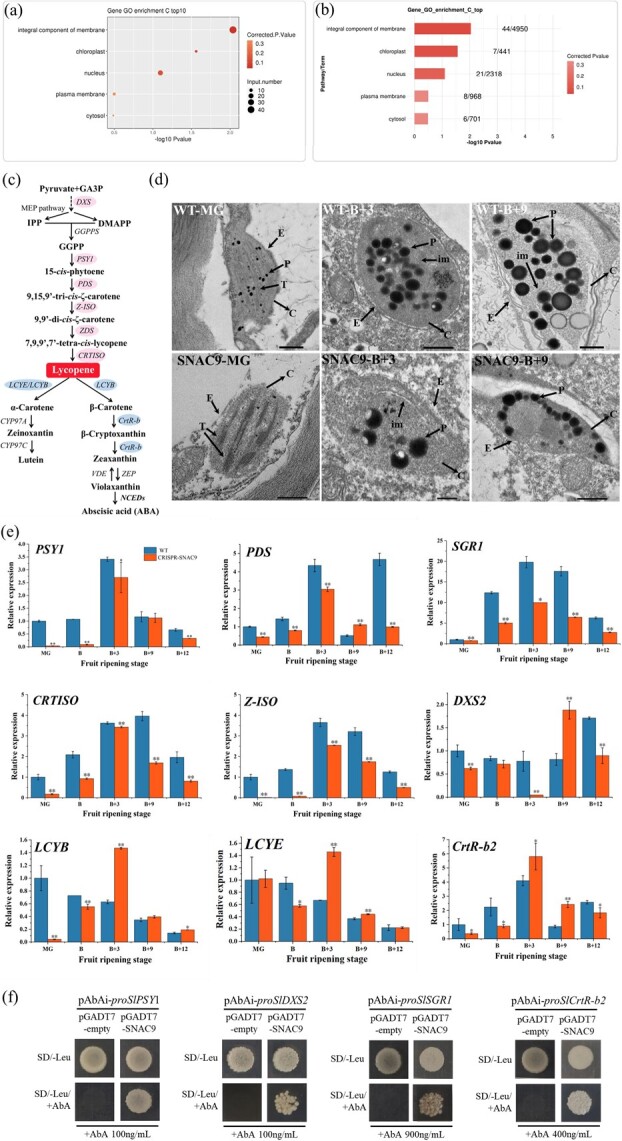
Effects of *SNAC9* knockout on critical genes of carotenoid metabolism and chromoplast. (a-b) GO enrichment analysis of SNAC9 samples combined with peak-related genes. (c) Carotenoid metabolic pathway. (d) Chloroplast in the MG period and chromoplast morphology in the B + 3 and B + 9 periods. E, plastid envelope; P, plastoglobuli; T, thylakoid grana; C, Chloroplast or chromoplast; im, rod-shaped internal membranes. (e) Relative expression levels of key genes for carotenoid metabolism. Tomato *Actin* gene was used for the internal reference gene. The bars represent the averages and standard deviations of 3 biological replicates. Asterisks show significant differences between WT and *SNAC9*-KO fruit (^*^P ≤ 0.05, ^**^P ≤ 0.01). (f) Characterization of DNA-binding activation ability of SNAC9 to pigment metabolism key gene promoter. The minimum AbA concentrations for inhibition of the *PSY1*, *DXS2*, *SGR1*, and *CrtR-b2* promoters were 100 ng/mL, 100 ng/mL, 900 ng/mL, and 400 ng/mL, respectively. The yeast one-hybrid (Y1H) experiment evaluated SNAC9 binding to the promoter segments of *PSY1*, *DXS2*, *SGR1*, and *CrtR-b2*. AbA: Aureobasidin A.

We compared the expression levels of pigment-related genes in fruits at various times to comprehend the function of SNAC9 in regulating pigment metabolism during fruit ripening at the molecular level (Fig. 5e).

The *DXS2* gene encoding 1-deoxy-D-xylose-5-phosphosynthase 2 was significantly higher in wild-type fruits than in the KO line at the green ripening stage, breaking color stage, and three days and twelve days after the breaking color stage. SNAC9 may play a role upstream of the carotenoid metabolic pathway. PSY1 and PDS are the critical dehydrogenases in lycopene biosynthesis (Fig. 5c). At each of the five phases of fruit ripening, the *PSY1* gene expression level in the *SNAC9* KO line was considerably lower than that in WT fruits. The variation trend was consistent with the *PDS* gene at the green ripening stage, breaking color stage, and 3 and 12 days after the breaking color stage. Three days after the breaker stage, the fruit was in the turning red stage, and lycopene began to accumulate. *CRTISO* encodes carotenoid isomerase, and *Z-ISO* encodes ζ-carotene isomerase, which promotes lycopene synthesis. The expression levels of *CRTISO* and *Z-ISO* in the WT group were significantly higher than those in the KO line at each of the five stages of fruit ripening, as shown in the image. STAY-GREEN (SGR) is a chlorophyll degradation factor. The deletion of *SNAC9* also affected *SGR1* expression. These results indicated that *SNAC9* gene knockout affects lycopene synthetase, which affects lycopene synthesis, consistent with fruit phenotype observations.

**Figure 6:**
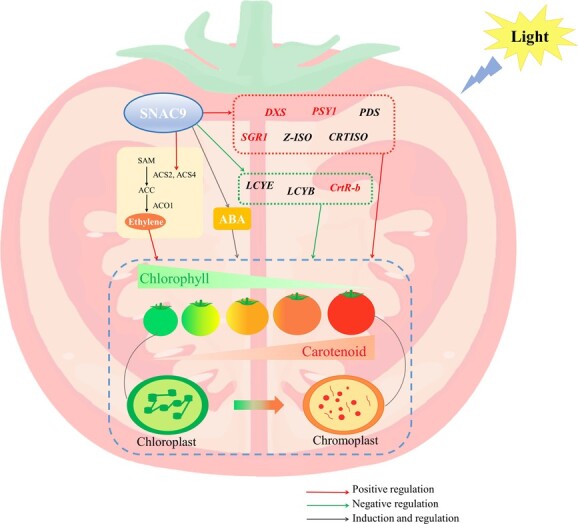
Regulation of SNAC9 on carotenoid metabolism in tomato fruits

The expression levels of *LCYE* (lycopene ε-cyclase) and *LCYB* (lycopene β-cyclase) were significantly higher than those of the wild type three days after the color-breaking stage, and lycopene was transformed into β-carotene. The β-carotene content in the KO line at the B + 12 stage was slightly higher, indicating that lycopene degradation was faster (Fig. 3g). Studies have shown that overexpression of *BCH1* and *BCH2* (*CrtR-b2*) in ripe tomatoes resulted in a decrease in total carotenoid concentrations, lycopene and β-carotene [38]. The expression of β-carotene hydroxylases (CrtR-b2) was lower than that of the wild type at the MG and breaker stages and higher than that of the wild type 3 days after the breaking stage, and β-carotene was transformed into zeaxanthin.

Chromoplasts are sites of carotenoid biosynthesis and storage [6]. The ultrastructure of the chromoplasts was investigated using transmission electron microscopy (TEM) (Fig. 5d). Small plastoglobuli were found in the WT pericarp at the MG stage, and thylakoids in chloroplasts began to decompose. However, the chloroplast structure of the KO line was intact. The membrane structure of WT fruits undulated, and the number of plastoglobuli increased, indicating that the transformation of chloroplasts to chromoplasts was complete at the B + 9 stage. The number of small plastoglobuli (P) in B + 3 and B + 9 plastids in KO fruit was reduced, and the plasma membrane structure was intact.

### SNAC9 activates the promoter activity of crucial genes in pigment metabolism

NAC proteins may also regulate fruit pigment metabolism through direct binding to the promoter regions of genes critical for pigment metabolism. Based on the above qRT–PCR results (Fig. 5e), we used yeast one-hybrid assays to verify SNAC9 regulation of pigment metabolism-related target genes. Screening of the minimum AbA concentration that inhibited the growth of the pAbAi-Bait strain showed that the AbA concentrations that inhibited the *PSY1*, *DXS2*, *SGR1*, and *CrtR-b2* promoters were 100 ng/mL, 100 ng/mL, 900 ng/mL and 400 ng/mL, respectively. In the absence of AbA, the yeast grew in both SD/−Leu dishes. When the AbA concentration was at the minimum inhibitory concentration of the promoter, colonies appeared in the petri dishes of the pGADT7-SNAC9 + *PSY1* promoter, pGADT7-SNAC9 + *DXS2* promoter, pGADT7-SNAC9 + *SGR1*, and pGADT7-SNAC9 + *CrtR-b2* promoter groups (Fig. 5f). The results of Y1H validated that the SNAC9 protein can bind to the *PSY1*, *DXS2*, *SGR1*, and *CrtR-b2* promoters. *PSY1*, *DXS2*, *SGR1*, and *CrtR-b2* can interact with SNAC9 at the DNA–protein level. These findings suggested that SNAC9 directly regulates the *PSY1*, *DXS2*, *SGR1*, and *CrtR-b2* genes to influence carotenoid accumulation and Chl degradation in tomato fruit.

## Discussion

In plants, carotenoids are crucial for signal transmission, hormone synthesis, pigmentation, and photosynthesis. They are also necessary for humans since they function as dietary antioxidants and precursors to the manufacture of vitamin A. The accumulation of carotenoids in the fruit gives it a bright orange, yellow or red color and is of ecological and agricultural importance. Due to the significance of carotenoids to plants and people, carotenoid metabolism in plants has been the subject of extensive research [6, 10]. Tomatoes are rich in carotenoids. The regulatory mechanism of tomato ripening is complex. In any highly redundant biological network, there may not be a single master regulator [29]. Recently, many TF families have been reported to impact fruit carotenoid metabolism, such as NAC, MADS-box [39], MYB [12], and bHLH [40]. The NAC family is one of the most prominent TF families. *SlNAC4* plays a positive role in carotenoid accumulation [17]. By influencing ethylene synthesis and carotenoid accumulation in *SlNAC1* overexpression lines, *SlNAC1* reduced fruit ripening [21]. The expression of the majority of carotenoid biosynthesis genes is increased when *FcrNAC22* is overexpressed in tomato and citrus fruits. This also speeds up the process of plastid conversion into chromoplasts and color change [41]. When CRISPR/Cas9 inactivates NOR-like1 function, ethylene production is significantly reduced, softening and chlorophyll loss are slowed, and lycopene accumulation is reduced [16]. Using VIGS technology and *SNAC*-silenced fruit, we previously examined the role of the transcription factors SNAC4 and SNAC9 during maturity. When *SNACs* were silenced, ripening was postponed along with decreased ethylene production, decreased lycopene, and yellow or orange fruits [42]. Compared with VIGS, CRISPR was used to knock out the *SNAC9* gene more thoroughly, and neither gene nor protein was expressed (Fig. 1d-e). Fruit color development was delayed in the *SNAC9* KO line. The chlorophyll degradation rate of the KO line was lower. Knockout of the *SNAC9* gene significantly affected carotenoid regulation, and lycopene and total carotenoid contents were significantly reduced. The study found that *SNAC9* regulates chloroplast to chromoplast transformation by regulating essential carotenoid metabolism genes in tomato ripening (Fig. 6).

We predicted that SNAC9 is closely associated with chloroplasts using ChIP (Fig. 5a-b). We found that chloroplast-to-plastid transformation was delayed (Fig. 5d). The results indicated that *SNAC9* positively regulated the synthesis of lycopene and negatively regulated the decomposition of lycopene (Fig. 5e). There are two carotenoid biosynthesis pathways: the isoprenoid and terpenoid pathways. The biosynthetic precursor isoprene pyrophosphate (IPP) is formed by the MEP pathway with five carbon atoms (Fig. 5c) [6]. DXS is the first step and critical regulatory enzyme of the MEP pathway in plants. The results suggest that *SNAC9* may interact with the *DXS2* gene and affect the isoprene pathway. The transcriptional abundance of *SNAC9* increased significantly with fruit ripening and peaked at the B + 3 stage. The expression patterns of *SNAC9* were consistent with those of *PSY1*, *SGR1*, and *Z-ISO*, suggesting that *SNAC9* may regulate these upstream genes. Here, we also verified that SNAC9 directly regulates the expression of the *PSY1*, *SGR1*, and *CrtR-b2* genes (Fig. 5f). Some studies have shown that *SGR*, *PSY*, *Z-ISO*, and *CRTISO* are all located in the chromoplast. Their high coexpression also indicates the probability of interacting with each other to form a complex [37]. The lack of LCYB activity leads to the accumulation of lycopene, which gives tomatoes their red color. *LCYE* encodes lycopene ε-cyclase, which transforms lycopene into α-carotene. *LCYB* encodes lycopene β-cyclase, which transforms lycopene into β-carotene or α-carotene. Compared with the control group, the contents of the two cyclases in the KO line increased significantly from 3 days after the color-breaking stage (Fig. 5e), which was also the reason why the fruit color in the KO line was lighter than that in the WT group, suggesting that *SNAC9* knockout could affect lycopene degradation by affecting the expression of the two cyclases.

Carotenoids are segregated and stored in carotenoid-lipoprotein substructures or plastoglobuli within the chromoplast [9]. Chromoplasts differentiated from chloroplasts are characterized by the complete degradation of chlorophyll and the disappearance of chloroplast thylakoids. This includes restructuring the endomembrane system and accumulating carotenoids in chloroplasts’ newly formed carotenoid isolation substructures [43]. When we found that the carotenoid content of the mutants was significantly reduced, the ultrastructure of the chromoplast was observed by transmission electron microscopy at the MG, B + 3, and B + 9 stages (Fig. 5d). At the MG stage, the chloroplast structure of the mutant fruit was relatively complete, the thylakoid grana structure was clear, and there were small plastoglobuli in the wild-type pericarp. The number of plastoglobuli in the B + 3 and B + 9 KO line plastids was reduced, and the plasma membrane structure was intact, consistent with the results of phenotypic observations and the determination of pigment-related indices. The results suggested that *SNAC9* knockout could affect the transformation of chromoplasts, affecting the color and carotenoid accumulation of fruits.

The function of NAC proteins under different biological stresses is complex. Some NAC proteins are targets of pathogen effectors, according to some studies [44], and some NAC proteins regulate immune signaling pathways and play a key role in plant immunity [45]. For example, SlNAP1 actively regulates defense against multiple stresses by accelerating GA inactivation and promoting the biosynthesis of ABA and salicylic acid (SA) [46]. *SlNAC35* and *SlSRN1* induce plant defense responses upon pathogen challenge through either jasmonic acid (JA) or SA signaling pathways [47]. *SlNAP2* (*SNAC9*) plays a complex role in ABA homeostasis establishment during fruit yield control and leaf senescence [31]. In transgenic tomato plants, inhibiting the expression of *SlNAP2* may delay leaf senescence but may prolong photosynthesis time in senescent tomato leaves. The ChIP results predicted that SNAC9 was involved in chloroplast metabolism (Fig. 5a-b). SGR proteins can mutually affect chlorophyll-degrading enzymes to impact chlorophyll degradation [37]. According to the findings, *SGR1* expression was much lower in the KO line than in the WT group (Fig. 5e), which suggested that Chl degradation slowed down, which was consistent with the shift in chlorophyll content (Fig. 3c). Additionally, research has demonstrated that overexpressing *SNAC9* can increase the expression of Chl-degrading genes such as *SlSGR1* [12, 48]. The DNA–protein level interaction between SNAC9 and *SGR1* was demonstrated using a yeast one-hybrid assay (Fig. 5f). These findings confirmed that SNAC9 directly controls *SGR1* to alter chlorophyll degradation. We also found that the plant height, true leaf number, and flower diameter of the mutant were significantly lower (Fig. 2). We hypothesize that *SNAC9* knockout might affect plant disease resistance by affecting plant growth. Future studies will examine the combined effects of *SNAC9* on plant defense and fruit physiological functions, its effects on plant disease resistance, and its regulation of fruit physiological functions to construct an integrated regulatory network of NAC transcription factors on plant growth and fruit ripening.

In conclusion, we utilized CRISPR/Cas9 to construct function-loss mutants of *SNAC9*. We established that SNAC9 regulates carotenoid metabolism during tomato ripening by comparing the fruit phenotype and relative gene expression levels for the carotenoid-related enzyme. We further found that *SNAC9* regulates critical genes (*PSY1*, *DXS2*, *SGR1*, and *CrtR-b2*) involved in pigment metabolism and then regulates the transformation of chloroplasts to chromoplasts. Meanwhile, *SNAC9* knockout also changed the expression levels of ethylene and ABA-critical genes. Our findings provide genetic material and an experimental basis for further exploring the mechanism by which NAC transcription factors regulate tomato fruit pigment metabolism and cell wall metabolism. It also lays the groundwork for raising the carotenoid content of crops to improve their nutritional worth and health benefits.

## Materials and methods

### Plant materials and growth conditions

Micro-Tom tomato seeds were submerged in distilled water at 25°C for 24 hours. Then, they were placed in flowerpots with a 7:1 ratio of soil to vermiculite. The tomatoes were grown in a greenhouse at 23 to 25°C with a 16 h/8 h light/dark cycle. Tomatoes grow at 70 to 85% relative humidity. To reliably track the ripening stages throughout fruit development, flowers were tagged at anthesis. The water supply (90% fruit water content) and potassium fertilizer supply (NPK) should be increased during the fruiting period. Fruits from wild-type and genetically modified lines were gathered at various stages of ripeness (MG, B, B + 3, B + 6, B + 9, and B + 12).

### Construction of the pYLCRISPR/Cas9Pubi-H-*SNAC9* vector and genetic transformation of tomato

The intermediate carrier pYLgRNA and dual carrier pYLCRISPR/Cas9Pubi-H were used. We designed the SNAC9 knockout targets using the online software toolkits CRISPR-GE (http://skl.scau.edu.cn/home/) and CRISPR-P 2.0 (http://crispr.hzau.edu.cn/CRISPR2/). The overlapping PCR technique was used to build the single-guide RNA (sgRNA) expression box, which was then combined into the pYLCRISPR/Cas9 vector by the Golden Gate cloning process. The colony was verified by Sanger sequencing after PCR. Using the *Agrobacterium* transformation technique, the genes for the Cas9 protein and sgRNA were delivered into Micro-Tom tomato cells. After successful transformation, the TransDirect Plant Tissue PCR Kit (TransGen Biotech, Beijing, China) and Cas9 gene-specific primers were used for PCR detection, and positive plants were screened. Target detection was performed on the positive vaccine. CRISPR-P and CRISPR-GE were used to predict off-target sites, and two off-target sites located in gene regions with high miss scores were selected for each target for detection. The primers utilized for vector construction are listed in Supplementary Table S1. Off-target site detection results are shown in Supplementary Table S2.

### Identification of gene target sequence mutations

After the mutant grew stably, the leaves of different parts were mixed and ground, and the genomes were extracted for corresponding target sequencing detection. Specific primers were designed for each target. Using 2 × Taq Mix, the fragment containing the target sequence was amplified for Sanger sequencing. ContigExpress, DSDecodeM, and SaDSDecode software were used to analyze the sequencing results. Target sequences of mutant and wild-type plants were compared. If two chromosomes have the same mutation, it was considered a homozygous mutation. Supplementary Table S1 provides a list of sequencing primers.

### Western blot analysis

The total protein of WT and SNAC9 mutant tomato leaves was extracted simultaneously, and the protein samples were denatured after the total protein content was determined. According to the molecular weight of the SNAC9 protein, the corresponding separation gel and concentrated gel were prepared using an SDS-PAGE rapid gel preparation kit, separated by electrophoresis, and transferred to a PVDF membrane. Mice were immunized with purified recombinant His-SNAC9 protein to obtain anti-SNAC9 polyclonal antibodies (Dynamiker Biotechnology Co., Ltd, Tianjin, China) and incubated with the target protein. Anti-β-actin mouse mAb (Zen-Bioscience, China) was used as the internal reference. The corresponding anti-mouse antibody (HRP-labeled goat-anti-mouse IgG, Zen-Bioscience, China) was selected as the secondary antibody. Following ECL staining, the pictures were examined under an optical microscope, and ImageJ software was used to assess the gray values.

### Firmness measurement

An FHM-1 hardness tester tested the tomato fruit at the symmetrical point on the equator of the fruit. Three circular areas were selected on the surface of the fruit with regular shape, and the pressure was pressed slowly at the marks. The pressure was stopped when the probe pierced the skin and entered the flesh, and the parameters were recorded in Newtons (N).

### Chromaticity analysis and soluble solids analysis

Fruit color was determined by a 3nh portable computer color meter. A standard whiteboard was used to calibrate the fruit L, a, and b values of the wild type and mutant in different periods. A PAL-1 (ATAGO, Japan) handheld refractometer was used to determine the content of soluble solids in tomato fruits.

### Pericarp microstructure analysis

Following previous methods [49] and modifying them, we rinsed the sample gently with distilled water to remove surface dirt and wiped it clean with a clean paper towel. Then, a clean and sterile scalpel was used to cut tomato peels (0.5 ~ 1.0 cm^2^) at the equator, and attention was given to minimize pulling during sampling to avoid mechanical damage and affect the observation results. The tomato skins were soaked with the fixative solution under an electron microscope and then fixed at room temperature, rinsed with 0.1 M PBS buffer (pH 7.4) three times for 15 min each to remove the fixative solution. The samples were immersed in 30%–50%–70%–80%–90%–95%–100%–100% ethanol for dehydration for 15 min each time and then dried with a vacuum freeze dryer for two hours. A Cressington 108 Autoion sputterer was used to apply gold to the samples. Next, the samples were examined using a Regulus 8100 SEM (Hitachi, Japan). 3.0 kV was set as the acceleration voltage.

### Carotenoid extraction and HPLC analysis

Tomato samples (0.5 g) were weighed in a conical flask and wrapped in tin foil. Additionally, 10 mL of a 2:1:1 volume ratio of n-hexane, methanol, and acetone was added, and the solvent mixture was agitated using magnetic force at 27°C for 30 minutes in the dark. After filtering the sample, collecting the filtrate, and extracting the residue twice, until it was colorless. The filtrate was collected three times. The organic and aqueous phases were separated using a liquid separation funnel. The organic phase containing the carotenoids was then collected and transferred to a flask with a flat bottom. After the solvent was evaporated by rotation, methanol and methyl tert-butyl ether (MTBE) were dissolved. Analysis was performed using a Waters HPLC e2695 system with a PDA diode array detector and a C18 column (250 mm × 4.6 mm, 5 μm). The flow rate was 1.0 mL/min, and the column temperature was 25°C. Methanol, acetonitrile, and MTBE are mobile phase components (27:23:50). External standards were used to quantify the amounts of lycopene, β-carotene, and lutein. The wavelengths are set to 471, 452, and 448 nm. Each sample underwent a minimum of three separate extractions.

### Ethylene measurement

According to a previously described method [42] with modifications, we exposed fruit to air at 20 ± 1°C for three h to counteract the effects of wound-induced ethylene. Then, we placed tomatoes in 20 mL gas-tight canisters and sealed them at 20 ± 1°C for three h. One milliliter of gas from the headspace sample was put into the gas chromatograph (GC-14C, Japan). Three parallel experiments were conducted.

### ABA HPLC analysis

According to a previously described method [42] with modifications, HPLC was used to determine the ABA content in tomato fruits. C18 (250 mm × 4.6 nm, 5 μm) is the chromatographic column. Water (solvent B) and acetonitrile (solvent A), both containing 0.05% acetic acid, served as the mobile phase. The sample volume was 5 μL, the column temperature was 30°C, and the flow rate was 0.8 mL/min. The detection wavelength was 262 nm.

### Total RNA isolation and real-time quantitative PCR (qRT–PCR)

The expression changes of *SNAC9*, pigment metabolism, and plant hormone-related genes after *SNAC9* gene knockout were analyzed and compared with ordinary fruits. The RNAprep Pure Plant Kit was used to extract the total RNA from tomato pulp, and the FastQuant RT Kit was used to create high-quality cDNA. SuperReal PreMix Plus (SYBR Green) was used for qRT–PCR in a Roche Light Cycler 480 instrument following the kit instructions. Each sample had three biological replicates, and the Formula 2^-ΔΔCT^ was used to calculate the relative expression levels of the genes. The primers are listed in Supplementary Table S3.

### Ultrastructure of chromoplast by transmission electron microscopy (TEM)

According to a previously described method [49, 50] with modifications, the sampling location of fresh tissue was determined. Samples were obtained between one and three minutes, and the size of the tissues was 1mm^3^. Then, the cut small tissue blocks were moved to an EP tube equipped with a new electron microscope fixative for further fixation and pumped by a vacuum pump until they sank to the bottom. After two hours at room temperature, they were fixed at 4°C. After rinsing, postfixation, dehydration at room temperature, osmotic embedding, polymerization, ultrathin sectioning, and staining, samples were observed with a JEM-1400 Flash TEM (JEOL, Tokyo, Japan).

### Yeast one-hybrid test

Referring to the experimental method of Yang et al. [24], and using tomato cDNA as a template, the full-length coding sequence of SNAC9 was amplified and ligated to the pGADT7 vector. To design primers, a 150 ~ 200 bp fragment of the promoter region of genes involved in pigment metabolism was used. Fruit genomic DNA was extracted using an Easy Pure Plant Genomic DNA Kit (CAT#: EE111–01, TransGen, Beijing, China.) and then utilized as a template for PCR amplification of cis-elements and linking to the pAbAi vector. The Y1H-Gold yeast strain was created by linearizing the resultant pAbAi-bait plasmid. By using pAbAi bait to turn the construct of AD-prey into the Y1H-Gold strain, and screened it on SD/−Leu/AbA over three to five days at 29°C. Supplemental Table S4 includes a list of the primer sequences.

### Statistical analysis

The averages and standard deviations (SD) were calculated from three replicates carried out in triplicates for each treatment. Single-factor analysis of variance was conducted using SPSS 28.0. When p < 0.05, the difference was considered statistically significant; when p < 0.01, it was considered extremely significant. The charts were created using Excel and Origin 2022 software.

## Acknowledgements

This work was supported by the National Natural Science Foundation of China, China [Grant No. 32072274 and 31871848]. We thank Yaoguang Liu (South China Agricultural University) and Hongliang Zhu (China Agricultural University) for helping with pYLgRNA and pYLCRISPR/Cas9Pubi-H vector.

## Author contributions

All the authors contributed to the discussion. Y.F. and H.X.K. conceived and designed the experiment; Y.F. performed experiments and data analyses and wrote the manuscript; H.X.K., H.Z.X., and E.C.W. were involved in revising the manuscript; S.Y. and Y.X.Z. collected the literature and revised the manuscript; X.Q.L. measured ethylene release; Y.Z.H. and J.Y.S. checked the language. All authors read and approved the final manuscript.

## Data availability

All data related to this manuscript are available in this paper and its supporting materials.

## Conflict of interest

None declared.

## Supplementary Data

Supplementary data is available at *Horticulture Research* online.

## Supplementary Material

Web_Material_uhad019
