# Supplementary figures and images for: CRISPR/Cas9-mediated SNAC9 mutants reveal the positive regulation of tomato ripening by SNAC9 and the mechanism of carotenoid metabolism regulation
Source: Hortic Res. 2023 Feb 10;10(4):uhad019. doi: 10.1093/hr/uhad019 (PMC10076210; doi:10.1093/hr/uhad019)

## Slide 1
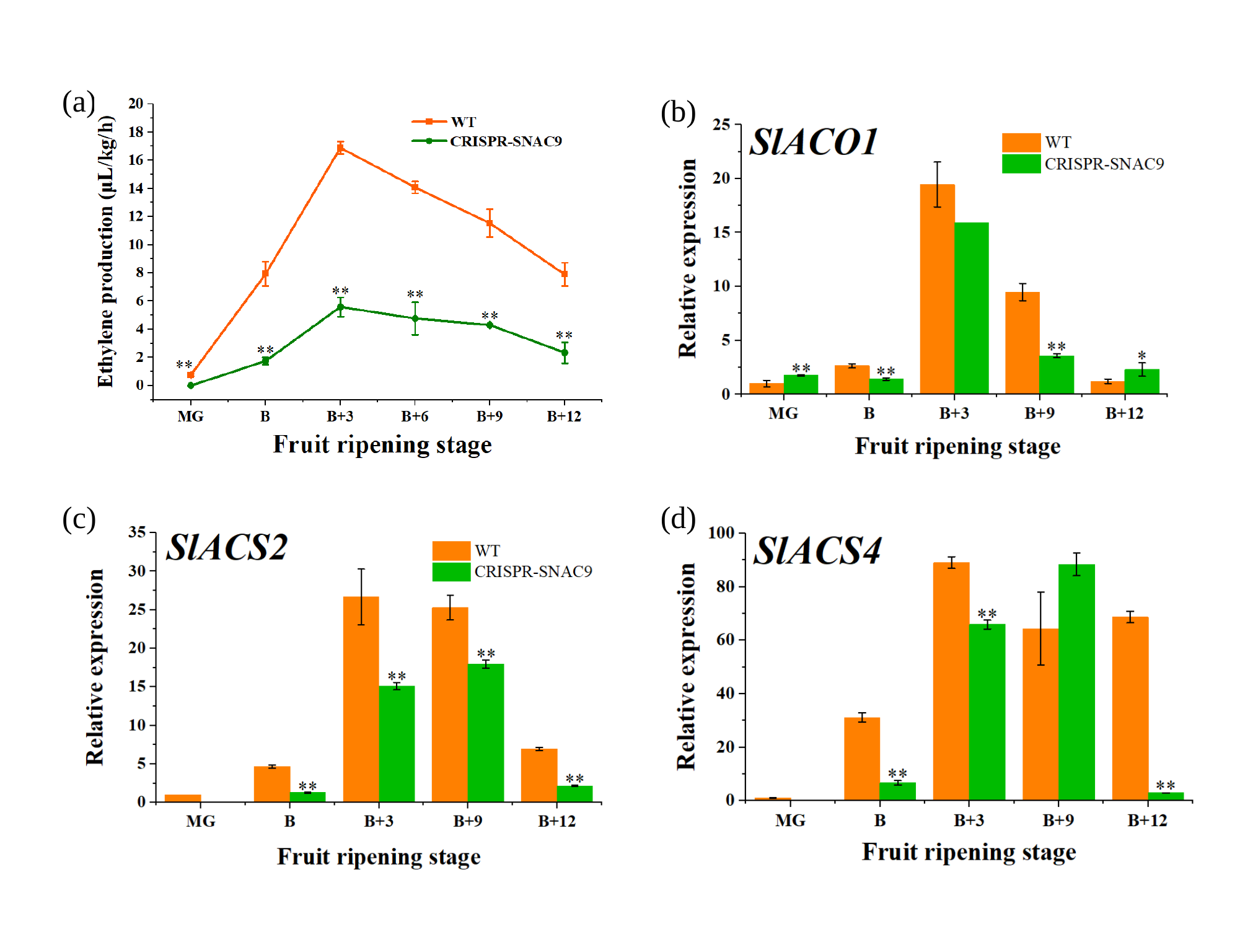

(a)
(b)
(c)
(d)

Supplement: Web_Material_uhad019 [file web_material_uhad019.zip › Figure. S1.pptx]

## Slide 1
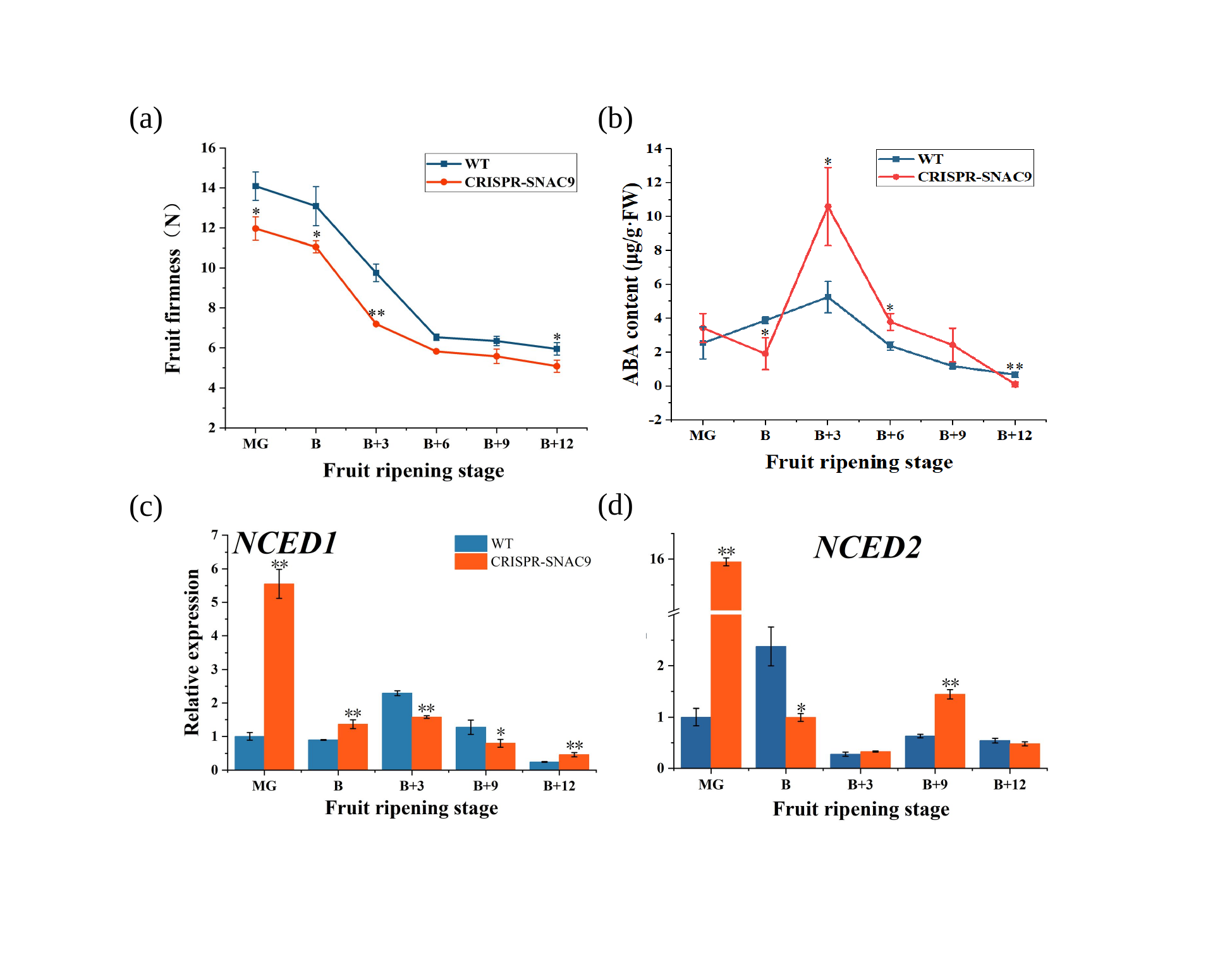

(b)
(a)
(d)
(c)

Supplement: Web_Material_uhad019 [file web_material_uhad019.zip › Figure. S2.pptx]
